# Role of diabetes in collateral status assessed in CT perfusion–derived dynamic CTA in anterior circulation stroke

**DOI:** 10.1007/s00234-021-02873-x

**Published:** 2021-12-09

**Authors:** Emilia Scheidecker, Benjamin Pereira-Zimmermann, Arne Potreck, Dominik F. Vollherbst, Markus A. Möhlenbruch, Christoph Gumbinger, Martin Bendszus, Christian Herweh, Fatih Seker

**Affiliations:** 1 Neuroradiology, Heidelberg University Hospital, Im Neuenheimer Feld 400, 69120 Heidelberg, Germany; 2 Radiology, University of Valparaiso, Valparaiso, Chile; 3 Neurology, Heidelberg University Hospital, Heidelberg, Germany

**Keywords:** Stroke, Collaterals, Diabetes, CTA

## Abstract

**Purpose:**

Diabetes is associated with vascular dysfunction potentially impairing collateral recruitment in acute ischemic stroke. This retrospective study aimed at analyzing the impact of diabetes on collateralization assessed on dynamic CTA.

**Methods:**

Collaterals were retrospectively assessed on CT perfusion–derived dynamic CTA according to the mCTA score by Menon in a cohort of patients with an acute occlusion of the M1 segment or carotid T. The extent of collateral circulation was related to the history of diabetes and to admission blood glucose and HbA1c levels.

**Results:**

Two hundred thirty-nine patients were included. The mCTA collateral score was similar in patients with diabetes (median 3, interquartile range 3–4) and without diabetes (median 4, interquartile range 3–4) (*P* = 0.823). Diabetes was similarly frequent in patients with good (18.8%), intermediate (16.1%), and poor collaterals (16.0%) (*P* = 0.355). HbA1c was non-significantly higher in patients with poor collaterals (6.3 ± 1.5) compared to patients with intermediate (6.0 ± 0.9) and good collaterals (5.8 ± 0.9) (*P* = 0.061). Blood glucose levels were significantly higher in patients with poor compared to good collaterals (mean 141.6 vs. 121.8 mg/dl, *P* = 0.045). However, there was no significant difference between good and intermediate collaterals (mean 121.8 vs. 129.5 mg/dl, *P* = 0.161) as well as between intermediate and poor collaterals (129.5 vs. 141.6 mg/dl, *P* = 0.161).

**Conclusion:**

There was no statistically significant difference among patients with good, intermediate, and poor collaterals regarding the presence of diabetes or HbA1c level on admission. However, stroke patients with poor collaterals tend to have higher blood glucose and HbA1c levels.

## Introduction

Acute ischemic stroke is the second most common cause of death [1]. Diabetic patients in particular have more than double the risk of ischemic stroke and admission hyperglycemia is associated with poor functional outcome [2]. It has not yet been clarified why diabetic patients tend to have poor clinical outcome. Based on animal experiments, several authors have discussed that diabetes might be associated with poor collaterals leading to poor outcome [3–5].

The role of collaterals in ischemic stroke has been studied in detail in the past few years [6]. Despite an intracranial large vessel occlusion, good collateral recruitment can temporarily protect ischemic brain tissue from extensive infarction and brain edema [7].

In contrast to the above-mentioned results of animal studies, clinical retrospective studies based on single-phase CT angiographies and single-phase collateral scores reported that diabetes is not associated with poor collaterals [8, 9]. Single-phase collateral scores, however, have been shown to be inferior compared to multiphase scores, because they do not depict the time delay and the spatial extent of collateralization in the late venous phase and, hence, might underestimate the actual collateralization [10, 11]. Multiphase scores also show better correlation with perfusion parameters and clinical outcome [12].

This retrospective study, therefore, aimed at analyzing the association of diabetes and collateral status using the multiphase CT angiography (mCTA) score by Menon et al. [13].

## Methods

### Patient selection

This is a retrospective observational cohort study. Institutional review board approval was obtained and informed consent was waived. All thrombectomy cases were prospectively collected in an institutional thrombectomy registry. All cases between April 2014 and March 2020 were screened retrospectively. Inclusion criteria for this analysis were (i) internal carotid artery occlusion reaching into the middle cerebral artery or M1 segment occlusion, and (ii) volume perfusion CT prior to thrombectomy. Patients with severe head movement during perfusion CT and insufficient intracranial contrast enhancement were excluded.

### CT perfusion

Dynamic CTA images were derived from CT perfusion data. The CT perfusion protocol has been described elsewhere [10]. Briefly, CT imaging was performed using a 64-multislice CT (SOMATOM Definition AS, Siemens, Erlangen, Germany) with an 8-cm *z*-axis coverage for CT perfusion. A contrast bolus of 36 ml Xenetix 350 (Guerbet, Sulzbach, Germany) followed by a saline flush of 20 ml at a flow rate of 6.0 ml/s was applied. Acquisition parameters for CT perfusion were 80 kV and 180 mAs. Acquisition duration was 44 s at a repetition rate of 1.5 s. Volume CT perfusion data were prospectively reconstructed with a slice thickness of 5 mm and archived in the institutional picture archiving and communication system.

### Collateral assessment

Collateral assessment was done by a reader with 5 years of experience in stroke imaging, who was blinded to clinical information including history of diabetes. Volume perfusion CT images were retrospectively manually retrieved from the picture archiving and communication system and postprocessed as dynamic CTA using the clinical application Dynamic angio in Syngo.Via version 7.3 (Siemens Healthcare, Erlangen, Germany) [14]. Collaterals were retrospectively assessed according to the mCTA collateral score by Menon et al. [10, 13, 15]. Briefly, this collateral score assesses both the spatial extent and the time delay of collaterals. Good collaterals were defined as mCTA score 4–5, intermediate collaterals were defined as 2–3, and poor collaterals were defined as 0–1 [13].

### Clinical data

Clinical data including comorbidities such as diabetes were routinely assessed during hospital stay and systematically documented in medical records by neurologists. Blood glucose and HbA1c levels were also routinely assessed on admission. These data were prospectively entered into our institutional thrombectomy registry.

### Statistical analysis

Statistical analysis was performed using R version 3.4.3 (R, Open Source). Collateral status was related to the history of diabetes and to blood glucose levels and HbA1c levels on admission. The three groups (good, intermediate, and poor collaterals) were compared using Kruskal–Wallis test. Post hoc Conover test with Benjamini–Hochberg correction was used for the comparison of two groups. A *P* value < 0.05 was considered statistically significant.

## Results

Two hundred thirty-nine patients were identified. Three patients were excluded due to severe head movement during CT perfusion acquisition. Six patients were excluded due to insufficient contrast enhancement during acquisition. In total, 230 patients were included in this study.

Of these, 127 patients (55.2%) were female. In 181 cases (78.7%), an M1 segment occlusion was present, and in 49 cases (21.3%), a carotid T occlusion was observed. Median National Institutes of Health Stroke Scale score was 16 (interquartile range 11–22). In 49 out of 230 patients (21.3%), type 2 diabetes was diagnosed. No other types of diabetes were present.

The mCTA collateral score was similar in patients with diabetes (median 3, interquartile range 3–4) and without diabetes (median 4, interquartile range 3–4) (*P* = 0.823). Diabetes was similarly frequent in patients with good (18.8%), intermediate (16.1%), and poor collaterals (16.0%) (*P* = 0.355) (Table 1).

**Table 1:** Comparison of baseline characteristics

	Good collaterals (*n* = 117)	Intermediate collaterals (*n* = 88)	Poor collaterals (*n* = 25)	*P* value^a^	*P* value^b^	*P* value^c^	*P* value^d^
Age, years, mean (SD)	74.4 (13.2)	73.5 (13.9)	74.1 (11.7)	0.870			
Female, *n* (%)	76 (65.0)	41 (46.6)	10 (40.0)	0.009	0.025	0.033	0.554
Premorbid mRS, median (IQR)	1 (0–2)	1 (0–2)	1 (1–2)	0.738			
Glucose, mg/dl, mean (SD)	121.8 (35.0)	129.5 (45.0)	141.6 (52.1)	0.040	0.161	0.045	0.161
HbA1c, mean (SD)	5.8 (0.9)	6.0 (0.9)	6.3 (1.5)	0.061			
Diabetes, *n* (%)	22 (18.8)	23 (16.1)	4 (16.0)	0.355			
Hypertension, *n* (%)	77 (65.8)	59 (67.0)	21 (84.0)	0.199			
CHD, *n* (%)	30 (25.6)	21 (23.9)	7 (28.0)	0.906			
Atrial fibrillation, *n* (%)	63 (53.8)	37 (42.0)	11 (44.0)	0.224			
Hyperlipidemia, *n* (%)	30 (25.6)	21 (23.9)	6 (24.0)	0.954			
Tandem lesion, *n* (%)	14 (12.0)	15 (14.0)	2 (8.0)	0.402			
Time onset to imaging, min, median (IQR)	206 (112–414)	183 (91–370)	122 (86–250)	0.053			

^a^Kruskal-Wallis test

^b^Post hoc Conover test between good and intermediate collaterals

^c^Post hoc Conover test between good and poor collaterals

^d^Post hoc Conover test between intermediate and poor collaterals

*CHD*, coronary heard disease; *IQR*, interquartile range; *mRS*, modified Rankin Scale; *SD*, standard deviation

HbA1c was non-significantly higher in patients with poor collaterals (mean 6.3 ± 1.5) compared to patients with intermediate (mean 6.0 ± 0.9) and good collaterals (mean 5.8 ± 0.9) (*P* = 0.061).

**Fig. 1:**
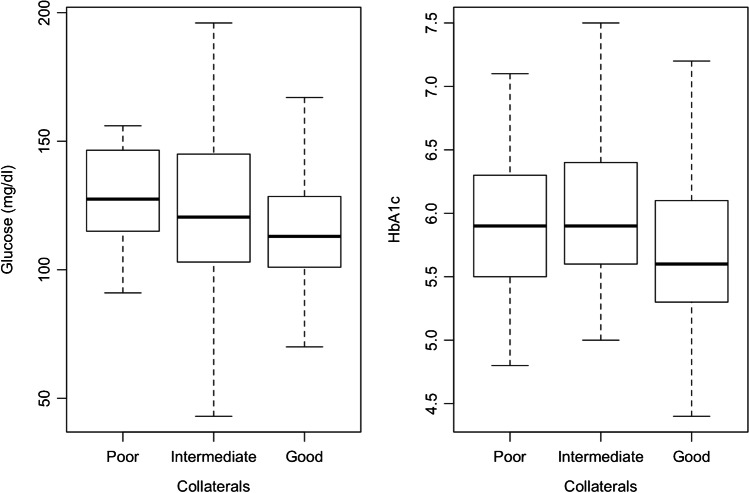
Blood glucose and HbA1c levels on admission in patients with good, intermediate, and poor collaterals. Although there is a trend towards higher glucose and HbA1c levels in poor collaterals, there is a distinct overlap of glucose levels among the groups

Blood glucose levels on admission were significantly higher in patients with poor compared to good collaterals (mean 141.6 vs. 121.8 mg/dl, *P* = 0.045). However, there was no significant difference regarding glucose levels between good and intermediate collaterals (mean 121.8 vs. 129.5 mg/dl, *P* = 0.161) as well as between intermediate and poor collaterals (129.5 vs. 141.6 mg/dl, *P* = 0.161). Although there was a trend towards higher glucose levels in poor collaterals, there was a distinct overlap of glucose levels among the groups (Fig. 1).

The proportion of female patients was higher in the group with good collaterals (65.0%) compared to intermediate (46.6%, *P* = 0.025) and poor collaterals (40.0%, *P* = 0.033). Comorbidities such as arterial hypertension and atrial fibrillation, however, were similarly frequent among the groups (Table 1).

## Discussion

There is a controversial debate regarding the influence of diabetes on collaterals in acute ischemic stroke. While results of animal studies indicate a strong association between diabetes and collateral status [3–5], clinical studies based on single-phase collateral assessment report to the contrary [8, 9, 16–18]. To our knowledge, this is the first study particularly analyzing the association of diabetes with collaterals based on dynamic/multiphase collateral assessment.

According to our results, presence of diabetes and HbA1c levels were similar among stroke patients with good, intermediate, and poor collaterals. HbA1c levels on admission were statistically non-significantly lower in patients with good collaterals compared to those with intermediate and poor collaterals. Possibly, statistical significance could be achieved with a higher sample size indicating that diabetic patients on a group level tend to have worse collaterals. However, even if these differences were statistically significant, both the mean values of HbA1c (5.8%, 6.0%, and 6.3%) and the overlap of values in all three groups (Fig. 1) demonstrate that these differences are of minor clinical relevance.

Our results based on the mCTA score confirm prior studies that utilized single-phase collateral scores. According to Christoforidis et al., blood glucose level was not associated with collaterals either. However, they included only 62 patients and used a collateral score that does not assess the spatial extent of collaterals [8]. Lazzaro et al. (*n* = 104) have reported no association of diabetes with collaterals using the Christoforidis score as well [9].

Borggrefe et al. have reported that glucose levels were elevated in diabetic patients with poor collaterals compared to diabetic patients with good collaterals according to the Tan score. However, there was no difference regarding collateral status in patients with and without diabetes [16].

According to a post hoc analysis of the MR CLEAN trial and MR CLEAN registry, high glucose levels were associated with poor collaterals on the Tan score. Diabetes was not associated with poor collaterals, though [17].

In a retrospective analysis of the Dutch Acute Stroke Study with 484 patients, the authors report that they could not find a relation between diabetes and collaterals. However, the authors used single-phase CT angiography and did not assess HbA1c [18].

Interestingly, studies based on animal experiments report the contrary. Biose et al. assessed brain perfusion using laser speckle contrast imaging in rats with middle cerebral artery occlusion and reported that hyperglycemia may impair the recruitment of cortical collaterals [5]. Akamatsu et al. assessed leptomeningeal collateral flow in mice using doppler optical coherence tomography and reported that diabetic mice exhibit impaired leptomeningeal collateral recruitment during stroke that was not attributable to acute hyperglycemia [3]. Yukami et al. report worse collateral recruitment in diabetic mice. Here, 14 days after common carotid artery occlusion, latex was injected into the left ventricle of the heart and photographs of the explanted brain were taken in order to evaluate collateralization [4].

Obviously, a variety of methods was used for collateral assessment in these animal models. However, none of these methods is comparable to CT angiography, which is used in daily clinical routine. The reduced comparability of the above-mentioned experimental methods with CT angiography may explain the divergence of results. Moreover, glucose metabolism, diabetes, and stroke pathogenesis may be different in animals compared to humans. Finally, animals may exhibit a different cerebral collateral system compared to humans.

Our study has several limitations, mainly due to its retrospective nature. Stroke patients who underwent CT perfusion, but who did not undergo thrombectomy, were not recorded in our registry and hence not included in this study. Also, patients who underwent thrombectomy without prior CT perfusion were not included in this study. Therefore, we cannot exclude a selection bias. Furthermore, it is not documented in our database whether patients were known to have diabetes on admission or whether diabetes was diagnosed during the hospital stay. Data on antidiabetic therapy was not available, either. We have therefore analyzed HbA1c levels, which are known to be a long-term indicator of blood glucose levels.

## Conclusions

There was no statistically significant difference among patients with good, intermediate, and poor collaterals regarding the presence of diabetes or HbA1c level on admission. However, patients with poor collaterals tend to have higher blood glucose and HbA1c levels on admission.

## Data Availability

Inquiries to the corresponding author.

## Abbreviation

mCTA: Multiphase CT angiography
